# Determinants of satisfaction 1 year after total hip arthroplasty: the role of expectations fulfilment

**DOI:** 10.1186/1471-2474-15-53

**Published:** 2014-02-24

**Authors:** Clémence Palazzo, Claire Jourdan, Stéphane Descamps, Rémi Nizard, Moussa Hamadouche, Philippe Anract, Stéphane Boisgard, Myriam Galvin, Philippe Ravaud, Serge Poiraudeau

**Affiliations:** 1 Service de rééducation et réadaptation de l’appareil locomoteur et des pathologies du rachis, AP-HP, Hôpital Cochin; PRES Sorbonne Paris Cité, Université Paris Descartes; U1153, INSERM, Paris, France; 2 Service de médecine physique et réadaptation, AP-HP, Hôpital Raymond Poincaré, Garches, Université Versailles St-Quentin, Guyancourt, France; 3 Service d’orthopédie, traumatologie, chirurgie plastique et reconstructive, CHU Gabriel Montpied; Université Claude Monnet, Clermont-Ferrand, France; 4 Service de chirurgie orthopédique et traumatologie, AP-HP, Hôpital Lariboisière; Université Paris Diderot, Paris, France; 5 Service d’orthopédie, AP-HP, Hôpital Cochin; PRES Sorbonne Paris Cité, Université Paris Descartes, Paris, France; 6 Centre d’épidémiologie clinique, AP-HP, Hôpital Hôtel Dieu; PRES Sorbonne Paris Cité, Université Paris Descartes, Paris, France

**Keywords:** Total hip arthroplasty, Expectations, Expectations’ fulfilment, Satisfaction, Outcome

## Abstract

**Background:**

Between 7% and 15% of patients are dissatisfied after total hip arthroplasty (THA). To assess predictors and postoperative determinants of satisfaction and expectation fulfilment one year after (THA).

**Methods:**

Before THA surgery, 132 patients from three tertiary care centres and their surgeons were interviewed to assess their expectations using the Hospital for Special Surgery Total Hip Replacement Expectations Survey (THR survey). One year after surgery, patients (n = 123) were contacted by phone to complete a questionnaire on expectation fulfilment (THR survey), satisfaction, functional outcome (Womac), and health-related quality of life (SF 12). Univariate and multivariate analyses were performed.

**Results:**

Preoperative predictors of satisfaction were a good mental wellbeing (adjusted OR 1.09 [1.02; 1.16], p = 0.01) and optimistic surgeons expectations (1.07 [1.01; 1.14], p = 0.02). The main postoperative determinant of satisfaction was the fulfilment of patient’s expectations (1.08 [1.04; 1.12], p < 0.001). Expectation fulfilment could be predicted before surgery by young age (regression coefficient −0.55 [−0.88; -0.21], p = 0.002), good physical function (−0.96 [−1.82; -0.10], p = 0.03) and good mental wellbeing (0.56 [0.14; 0.99], p = 0.01). Postoperative determinants of expectation fulfilment were functional outcome (−2.10 [−2.79; -1.42], p <0.001) and pain relief (−14.83 [−22.38; -7.29], p < 0.001).

**Conclusion:**

To improve patient satisfaction after THA, patients’ expectations and their fulfilment need to be carefully addressed. Patients with low mental wellbeing or physical function should be identified and specifically informed on expected surgical outcome. Surgeons’ expectations are predictive of satisfaction and information should aim to lower discrepancy between surgeons’ and patients’ expectations.

## Background

Hip osteoarthritis is a frequent [1-4] and disabling [1,4] disease, and its prevalence is increasing [1]. Total hip arthroplasty (THA) is currently the most efficient procedure to reduce disability for individuals with end-stage hip osteoarthritis, once conservative therapies to manage symptoms have been exhausted [5]. It provides excellent pain relief and improves functional status and wellbeing [6,7]. However, 7% to 15% of patients are dissatisfied after surgery [8,9]. Considering estimations for 2030 which predict a 174% increase in total hip replacements in the United States in comparison with 2005 [10], 572 000 patients could undergo a THA each year, and 35 000 to 75 000 could be dissatisfied. Important technical progresses have already been made in THA, so future progress in this field might not significantly impact patient satisfaction. An emerging area of research lies in the identification of determinants of patient dissatisfaction [9,11,12], which may offer new improvement perspectives in quality of care.

Previous studies have reported that satisfaction with THA was associated with limp absence, pain relief and function improvement [9,11-13]. Several preoperative risk factors for dissatisfaction have been identified: higher age, female gender, co-morbidities, associated conditions affecting walking capacity, mental distress, higher pain, and lower socioeconomic status [9,14,15]. There is however no evidence for a strong influence of any of these factors.

Other studies reported the role of patients’ expectations in postoperative satisfaction [13,16,17]. Three theoretical models have been suggested to explain the relationship between expectations and satisfaction [11]. The first model suggests that optimistic expectations could be per se a predictor of a higher ulterior satisfaction [18,19]. According to the second model, the fulfilment of patients’ expectations, independently of their preoperative level, is the main determinant of satisfaction [12,13,20]. The third theory assumes that postoperative symptoms and function determine patients’ satisfaction, regardless of their prior expectations [11,17,21,22].

While several authors adressed patients’ expectations and their consequences [13,22], data on surgeons’ expectations are scarce. In a previous work, we found that surgeons and patients did not agree on what to expect, particularly for some activities such as sport [23]. Surgeons rated their expectations according to preoperative function. For patients with severe disability, their expectations were more pessimistic than their patients’. However, the accuracy of surgeons’ expectations in predicting postoperative satisfaction of patients has not been studied.

The first objective of this study was to identify preoperative predictors and postoperative determinants of satisfaction one year after THA, including patients’ and surgeons’ expectations, and the second was to identify predictors and determinants of expectation fulfilment.

## Methods

### Participants

The present work is a longitudinal telephone-based follow up of a sample of patients who participated in a previous study [23]. Patients on waiting list for primary THA were recruited between January and June 2009 by 16 surgeons in 3 French tertiary care orthopaedic centres (APHP Lariboisière Hospital, Paris, APHP Cochin Hospital, Paris, and Gabriel Montpied Hospital, Clermont-Ferrand). Both patients and surgeons were contacted separately before surgery to answer a questionnaire concerning their expectations. Patients’ assessments also included symptoms, functional limitations, and psychological wellbeing. The preoperative sample included 132 patients. Indications for surgery were mostly primary or secondary hip osteoarthritis (82%), and avascular necrosis (12%).

Patients were included in the current postoperative study if THA had not been cancelled, if they could be contacted for a follow-up interview and still willing to participate.

The trial protocol was approved by the APHP Bichat hospital Research Ethics Committee (IRB00006477), and all participants had given written informed consent for the study.

### Evaluation

Patients were contacted by phone at a median delay of 379 days after surgery (inter-quartile range = 311–421), by two independent assessors (CJ and CP). The interview was standardized.

Since preoperative expectations of patients and surgeons had been assessed in the previous study using the Hospital for Special Surgery Total Hip Replacement Expectations Survey (THR Survey) [13,17,24,25], adapted to French by back translation [26], fulfilment of patients’ expectations for THA were assessed using the same tool [13,22]. This scale rates expectations of THA in eighteen domains, regarding symptom relief, improvement in physical function and in psychological wellbeing. The main question was adapted to assess the improvement that patients obtained from the surgery in each domain: “To what extent have you obtained a relief or improvement as a result of THA in the following areas?” (from 0: not at all; to 4: completely) [13,22]. The answer “not at all” (scoring 0) was separated from the answer “this question does not apply” (scoring 5). The global postoperative score, called postoperative THR survey, was calculated by summing the scores of all applicable items, and transformed by the formula: (sum/4*number of applicable items)*100, to obtain scores ranging from 0 to 100 [25]. Patients were not informed of their preoperative answers during the follow-up assessment. Items which were not applicable before surgery were regarded as equally unapplicable after surgery. The fulfilment score was defined as the percentage of applicable items for which preoperative patients’ (or surgeons’) expectations were fulfilled, meaning that postoperative rating of this item (ie obtained improvement) was equal or higher than its preoperative rating (ie expected improvement).

Patients were asked to rate their overall satisfaction with surgery using the following question [13]: “If you were to spend the rest of your life with your hip symptoms just the way they have been in the last twenty-four hours, how would you feel?”. This question was validated for general well-being assessment [13,27], and has been used previously in post-THA satisfaction assessment [13]. The seven response options ranged from “delighted” to “terrible”. Patients were classified in 2 groups, depending on whether they were satisfied with the surgery (including: “delighted”, “very satisfied” and “mostly satisfied”) or dissatisfied (including: “mixed feelings”, “mostly dissatisfied”, “unhappy” and “I feel terrible”).

Functional evaluation used the short 8-item Western Ontario and Mac Master Universities (WOMAC) functional subscale [28,29], which ranges from 0 (no disability) to 32 (extreme disability). Health related quality of life was assessed by the medical outcome study Short Form-12 (SF-12) [30,31], which includes a physical (SF-12 PCS) and a mental section (SF-12 MCS), both ranging from 0 to 100, and for which higher scores indicate better quality of life. Complications and presence of a limp were recorded. Pain and trust in surgeon were rated using a visual scale (from 0 to 100) before surgery. After surgery, pain was assessed by the two first questions of the THR survey (total relief of hip day and night pain *versus* residual hip pain).

Demographic characteristics included gender, professional category, and marital status. Health status evaluation included age, Body Mass Index (inferred from patients’ reports of height and weight). Co-morbidities were measured using the Charlson Comorbidity Index (no relevant comorbidity versus one or more co-morbidities). A history of ipsilateral hip arthroplasty was also recorded.

### Statistical analysis

Data was summarized as mean and standard deviation (SD) for continuous variables, and as count and percentage for categorical variables. Satisfied and dissatisfied patients were compared using chi-squared or Fisher’s exact tests for categorical variables, and Student’s t-tests or Mann–Whitney tests for continuous variables. Correlation between patient’s fulfilment score and continuous variables was assessed by Pearson’s correlation coefficient. An ANOVA was used to compare patients’ fulfilment score in the three centres.

Nonlinear mixed effect models (with centre as random effect) were computed to explain patients’ satisfaction through socio-demographic data, pre- and postoperative factors (analysed as fixed effects). Variables which were associated with satisfaction in univariate analyses at the 0.2 level were initially included; the best model was selected using second-order Akaike information criterion. Two different models were computed. The first model (called model 1) was predictive; it aimed to predict satisfaction after THA and only included preoperative parameters (= predictors) as covariables. The second model (called model 2) was explanatory; it aimed to explore which factors, among pre- and postoperative parameters, were most associated with satisfaction. Results were expressed as adjusted odds ratios (OR) and 95% confidence intervals (CI).

Two linear mixed effect models were also computed to assess determinants of the fulfilment of patient’s expectations (patients’ fulfilment score). The first model (model 1) was predictive and only included preoperative parameters. The second model (called model 2) was explanatory and included pre- and postoperative parameters. Results were expressed as regression coefficients and their 95% confidence intervals (CI), and the pseudo R^2^ was calculated to assess the proportion of variance explained by the model [32].

As missing data were scarce (6.8%), analyses were realized on complete data.

For univariate and multivariate tests, two-sided p-values ≤ 0.05 were considered to be significant.

Analyses were performed with SAS version 9.2 (SAS Institute, Cary, North Carolina) and R version 2.14.0 (R Foundation for Statistical Computing, Vienna, Austria).

## Results

Among the 132 patients of the preoperative study, 123 received the follow up evaluation: 60 patients from Cochin hospital (APHP, Paris), 46 from Lariboisière hospital (APHP, Paris) and 17 from Gabriel Montpied hospital (Clermont-Ferrand). Nine patients (6.8%) were not evaluated at follow-up (respectively 3, 4 and 2 from each center): 2 had not been operated, 6 were impossible to contact, and 1 had presented a hip fracture before surgery. These patients did not significantly differ from the others, excepted for a younger age (mean = 53.8 ± 13.7 years old).

### Population characteristics and postoperative outcome

Preoperative characteristics and outcome of patients are summarized in Table 1. Functional status improved after surgery. Nine complications (7.3%) were observed: 4 dislocations, 4 fractures (3 during surgery and 1 from a fall the day after surgery), 1 severe cutaneous reaction to the bandage. Twenty-one patients (17%) reported a residual limp.

**Table 1:** Pre- and postoperative characteristics of the study population (n = 123)

		**Preoperative characteristics**	**Postoperative characteristics**
Age (years)	(mean ± SD)	63.5 ± 13.5	-
Gender: man	count (%)	62 (50.4%)	-
BMI (kg/m^2^)	(mean ± SD)	25.8 ± 4.1	-
Number of co-morbidities	(mean ± SD)	0.9 ± 1.3	-
WOMAC	(mean ± SD)	18.6 ± 5.5	5.5 ± 6.1
SF-12 PCS	(mean ± SD)	32.4 ± 7.9	45.8 ± 6.8
SF-12 MCS	(mean ± SD)	48.4 ± 11.1	44.1 ± 5.7
Patients’ THR Survey	(mean ± SD)	90.7 ± 11.6	84.1 ± 20.9
Surgeons’ THR Survey	(mean ± SD)	90.1 ± 11.3	-

WOMAC: 8-item WOMAC functional subscale; SF-12 PCS: SF-12 Physical Component Score; SF-12 MCS: SF-12 Mental Component Score; THR survey: Hospital for Special Surgery Total Hip Replacement Expectations; Co-morbidities were measured using the Charlson Comorbidity Index.

### Preoperative predictors and post-operative determinants of satisfaction

Overall, 91.9% (n = 113) of patients were satisfied after THA (52 were delighted, 39 very satisfied and 22 mostly satisfied), and 8.1% (n = 10) were dissatisfied (5 had mixed feelings, 4 were unhappy and 1 felt terrible). In univariate analyses (Table 2), patients who were satisfied were younger and had a higher psychological wellbeing before surgery. In the group of satisfied patients, surgeons’ expectations had been more optimistic, and patients’ expectations had a non-significant higher trend. The difference between the patients’ and his surgeons’ expectations score was not significantly associated with satisfaction. Postoperative outcome was strongly associated with satisfaction: WOMAC was lower and physical component of SF-12 higher for satisfied patients; residual limp and pain were less frequent, and patients’ and surgeons’ expectations more frequently fulfilled.

**Table 2:** Univariate analyses of factors associated with satisfaction

		**Satisfied (n = 113)**	**Dissatisfied (n = 10)**	**p-value**
**Socio-demographic and medical data**				
Centre	count (%)			
1		51 (86.7%)	8 (13.3%)	
2		46 (97.9%)	1 (2.1%)	0.08
3		16 (94.1%)	1 (5.9%)	
Gender: man	count (%)	59 (52.2%)	3 (30.0%)	0.21
Co-morbidity>0	count (%)	51 (45.1%)	3 (8.6%)	0.51
Age	(mean ± SD)	62.8 ± 12.8	70.9 ± 18.5	0.02*
**Preoperative parameters**				
Pain	(mean ± SD)	7.5 ± 1.9	6.7 ± 2.0	0.24
WOMAC	(mean ± SD)	18.6 ± 5.7	19.1 ± 3.7	0.80
SF-12 PCS	(mean ± SD)	32.3 ± 8.0	34.1 ± 7.6	0.42
SF-12 MCS	(mean ± SD)	49.3 ± 10.7	38.5 ± 11.5	0.01*
THR Survey patients	(mean ± SD)	90.5 ± 11.4	86.4 ± 13.5	0.33
THR Survey surgeons	(mean ± SD)	91.3 ± 11.3	84.7 ± 10.0	0.04*
THR Survey patients - THR Survey surgeons	(mean ± SD)	- 0.8 ± 14.6	1.7 ± 17.1	0.59
Trust in surgeon	(mean ± SD)	9.2 ± 1.0	8.9 ± 1.2	0.35
**Postoperative parameters**				
Time from surgery (days)	(mean ± SD)	365.9 ± 76.9	337.8 ± 74.5	0.27
Residual pain	count (%)	26 (23.0%)	7 (70.0%)	<0.001*
Limp	count (%)	15 (13.3%)	6 (60.0%)	<0.001*
Complication	count (%)	8 (7.1%)	1 (10.0%)	0.59
WOMAC	(mean ± SD)	4.6 ± 5.1	15.8 ± 7.4	<0.001*
SF-12 PCS	(mean ± SD)	46.4 ± 6.5	39.4 ± 7.4	0.007*
SF-12 MCS	(mean ± SD)	44.2 ± 5.7	42.5 ± 5.8	0.34
Patients’ fulfilment score	(mean ± SD)	77.6 ± 23.7	22.4 ± 24.3	<0.001*
Surgeons’ fulfilment score	(mean ± SD)	77.4 ± 23.2	23.9 ± 28.0	<0.001*

WOMAC: 8-item WOMAC functional subscale; SF-12 PCS: SF-12 Physical component score; SF-12 MCS: SF-12 Mental component score; THR Survey: Hospital for special surgery total hip replacement expectations survey; Co-morbidities were measured using the Charlson comorbidity index. *p<0.05.

In multivariate analyses (Table 3), the preoperative predictors of satisfaction were psychological wellbeing and surgeons’ expectations in model 1. In model 2, which included postoperative variables, the only parameter selected in the final model was patients’ expectations fulfilment score.

**Table 3:** Multivariate analyses of factors associated with satisfaction

	**Model 1**		**Model 2**	
	**Preoperative factors**		**Pre- and postoperative factors**	
	**Adjusted OR [95% CI]**	**p value**	**Adjusted OR [95% CI]**	**p value**
Age	0.95 [0.89; 1.02]	0.13	NS	
Preoperative SF-12 MCS	1.09 [1.02; 1.16]	0.01*	NS	
Preoperative THR survey surgeon	1.07 [1.01; 1.14]	0.02*	NS	
Patients’ fulfilment score	-	-	1.08 [1.04; 1.12]	<0.001*

SF-12 MCS: SF-12 Mental component score; THR Survey: Hospital for special surgery total hip replacement expectations survey; Patients’ fulfilment score: mean percentage of individual expectations that have been fulfilled; NS: not selected in the final model; *p<0.05.

### Preoperative predictors and post-operative determinants of expectations fulfilment

**Figure 1:**
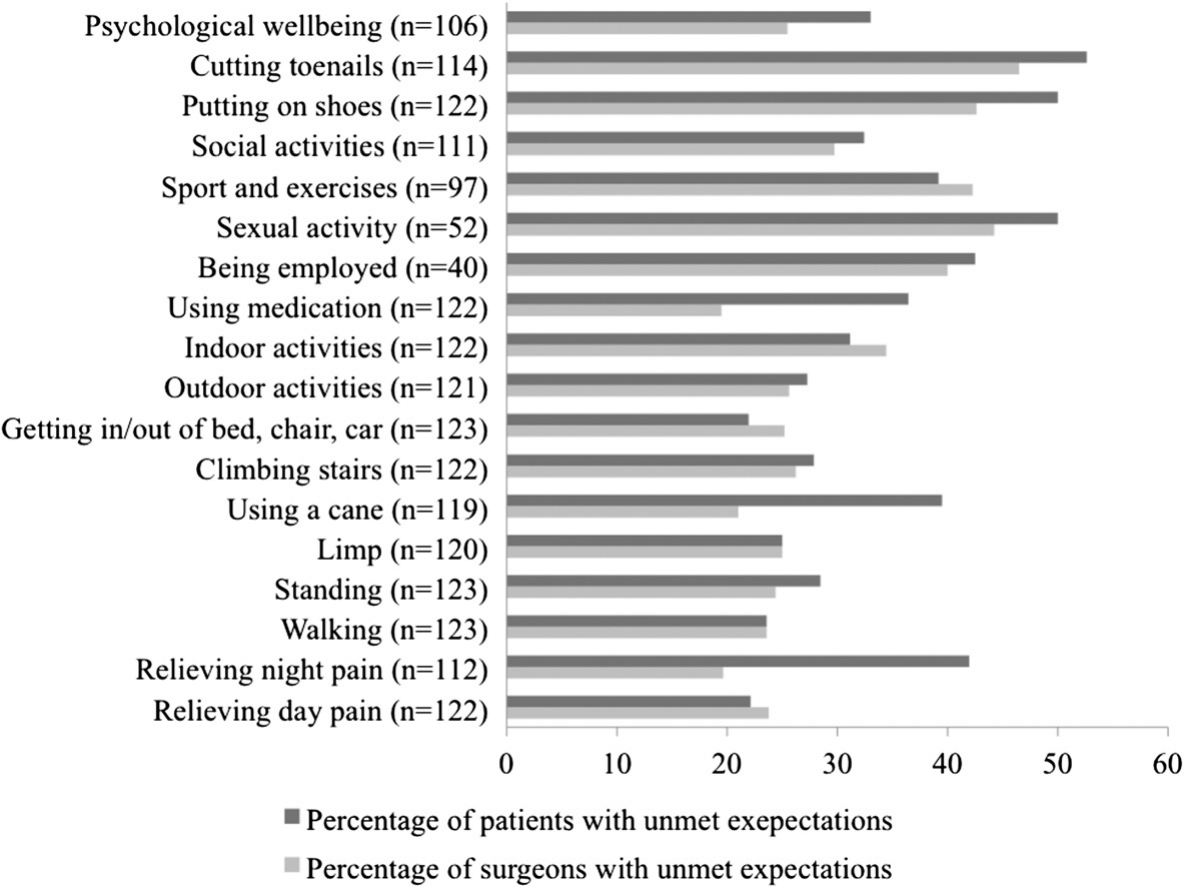
Frequency of patients and surgeons for which expectations were unmet regarding the 18 items of the THR survey (“n = …” corresponds to the number of applicable items).

The average percentage of fulfilled expectations was similar for patients and surgeons (73.1 ± 28.1 and 73.0 ± 28.3, respectively). Considering the fulfilment of expectations item by item (Figure 1), both surgeons’ and patients’ expectations were frequently unmet for cutting toenails (53% of patients and 47% of surgeons had unmet expectations in this domain), putting on shoes (50% and 43%), improving sexual activity (50% and 44%), improving sport and exercises (39% and 42%), and being employed (43% and 40%). There were higher patients-surgeons discrepancies for other items such as relieving night pain (42% of patients and 20% of surgeons had unmet expectations in this domain), or getting rid of cane (40% and 21%).

In univariate analyses (Table 4), patients’ fulfilment score strongly correlated with functional result (rho = − 0.71 for WOMAC). Expectations’ fulfilment was lower in case of residual pain or limp. Considering pre-operative variables, patients’ fulfilment score was significantly higher for men, younger patients, patients who experienced little pain and had no disability before surgery, or a better psychological wellbeing. Patients’ fulfilment score differed significantly according to care centres.

**Table 4:** Univariate analyses of factors associated with the fulfilment of patients’ expectations

		**Patients’ fulfilment score: mean ± SD**	**Rho [95% CI]**	**p-value**
**Socio-demographic and medical data**				
Age		–	– 0.30 [−0.45; –0.13]	<0.001*
Trust in surgeon		–	0.03 [−0.15; –0.21]	0.74
Centre	1	69.70 ± 27.66	–	<0.001*
	2	80.00 ± 23.10	–	
	3	66.62 ± 38.28	–	
Gender	Men	81.86 ± 20.62	–	<0.001*
	Women	64.23 ± 31.80	–	
Co–morbidity	No	71.96 ± 29.19	–	
	Yes	74.60 ± 26.79	–	
**Preoperative parameters**				
Pain		–	– 0.20 [−0.36; –0.02]	0.03*
WOMAC		–	– 0.27 [−0.42; –0.09]	0.003*
SF–12 PCS		–	0.15 [−0.03; 0.32]	0.10
SF–12 MCS		–	0.30 [0.13; 0.45]	<0.001*
**Postoperative parameters**				
Time from surgery (days)		–	0.10 [−0.08; 0.28]	0.27
Residual pain	Yes	80.77 ± 22.19	–	<0.001*
	No	52.26 ± 31.98	–	
Limp	Yes	80.70 ± 20.26	–	<0.001*
	No	36.30 ± 31.92	–	
Complication	Yes	74.09 ± 27.94	–	0.10
	No	62.16 ± 28.75	–	
WOMAC		–	– 0.71 [−0.79 ;–0.62]	<0.001*
SF–12 PCS		–	0.38 [0.22; 0.52]	<0.001*
SF–12 MCS		–	0.08 [−0.10; 0.25]	0.39

WOMAC: 8–item WOMAC functional subscale; SF–12 PCS: SF–12 Physical component score; SF–12 MCS: SF–12 Mental component score; Patients’ fulfilment score: mean percentage of individual expectations that have been fulfilled; Rho = Pearson’s correlation coefficient; Co–morbidities were measured using the Charlson comorbidity index. *p<0.05.

Results of multivariate analyses are presented in Table 5. Preoperative predictors of patient’s fulfilment score were age, preoperative WOMAC and SF-12 MCS (see Model 1). Model 1 explained 22% of the variance of patient’s fulfilment score. After including postoperative factors (Model 2), postoperative WOMAC, a residual pain and a residual limp were the variables significantly associated with patient’s fulfilment score; this second model explained 61% of the variance of patient’s fulfilment score.

**Table 5:** Multivariate analyses of factors associated with the fulfilment of patients’ expectations

	**Model 1 Preoperative factors**		**Model 2 Pre- and postoperative factors**	
	**Regression coefficient [95% CI]**	**p value**	**Regression coefficient [95% CI]**	**p value**
Age	−0.55 [−0.88; −0.21]	0.002*	−0.23 [−0.48; 0.02]	0.07
Preoperative pain	−0.45 [−17.0; 71.20]	0.73	NS	−
Preoperative WOMAC	−0.96 [−1.82; −0.10]	0.03*	NS	−
Preoperative SF-12 MCS	0.56 [0.14; 0.99]	0.01*	NS	−
Postoperative WOMAC	−	−	−2.10 [−2.79; −1.42]	<0.001*
Postoperative SF-12 PCS	−	−	NS	−
Residual limp	−	−	−18.05 [−28.53; −7.57]	0.001*
Residual pain	−	−	−14.83 [−22.38; −7.29]	<0.001*
**Pseudo R**^**2**^	**0.22**		**0.61**	

WOMAC: 8-item WOMAC functional subscale; SF-12 MCS: SF-12 Mental component score; SF-12. PCS: SF-12 Physical component score; NS: not selected in the final model. *p<0.05.

**Figure 2:**
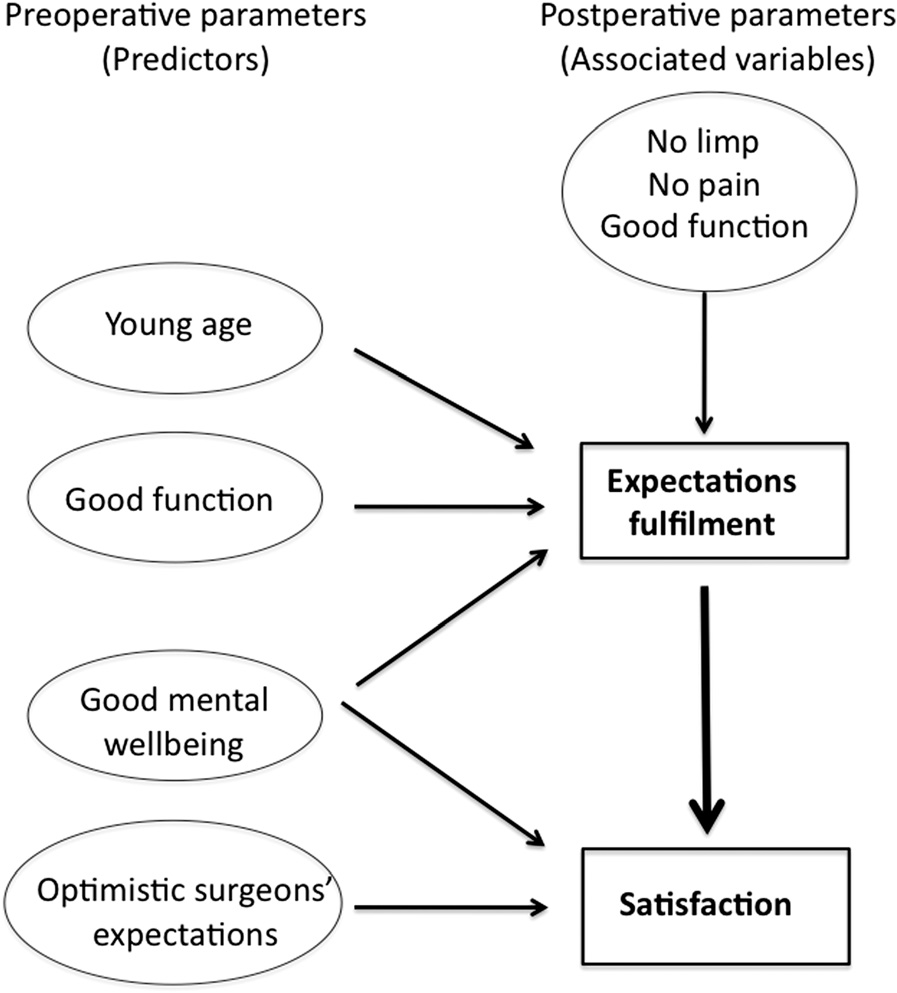
Predictors and variables associated with satisfaction and expectations fulfilment (it only includes the relationships explored in this study).

The Figure 2 summarizes the pre and post-operatives determinants of satisfaction and expectations fulfilment revealed by the above-mentioned models.

## Discussion

This study confirmed the excellent outcomes provided by THA [9,22]. Preoperative predictors of satisfaction were a good mental wellbeing and optimistic surgeons expectations. The main postoperative determinant of satisfaction was the fulfilment of patient’s expectations. Expectation fulfilment could be predicted before surgery by young age, good physical function and good mental wellbeing. Postoperative determinants of expectation fulfilment were functional outcome and pain relief.

### Predictors of dissatisfaction and implications on pre-surgical patient management

Surgeons, who rated their expectations according to preoperative function in our previous findings [23], were more reliable in predicting postoperative outcome than patients, who were more influenced by mental wellbeing and trust in their surgeon [23]. Pre-surgical patient information given by surgeons would thus need to be more specific on expected outcome. Particular attention should be given regarding patients with worse preoperative mental wellbeing, as this subgroup of patients were at higher risk of dissatisfaction after surgery, in our findings and in previous literature reports [14,22,33].

After adjustment for pre and post-surgical variables, fulfilment of patients’ expectations was the only significant determinant of satisfaction. And although patients’ preoperative expectations tended to be more optimistic in the group who was later satisfied, this difference was not significant. These results support the hypothesis that it is the fulfilment of expectations which determines satisfaction [12,13,20,22], independently of the preoperative expectation level [17]. To obtain higher satisfaction in THA, an important issue would then be to anticipate expectation fulfilment, by targeting patients with unrealistic expectations.

We did not find any significant association between co-morbidities and postoperative satisfaction, contradicting previous findings [7,9]. This might be due to the low number of co-morbidities in our sample, compared to reports from other studies [9]. Moreover, we did not specifically assess musculoskeletal diseases, such as other joint arthritis or back pain, which could be omitted in the reporting of diseases [34], and may influence postoperative satisfaction [7,9].

While other studies showed that patients preoperative function predicted outcome and satisfaction [35-37], the preoperative Womac score was not selected in our regression models. This could be explained by the adjustment on surgeons’ expectations in our study; surgeons rated their expectations according to preoperative function, as previously discussed.

### Role of patients’ expectations and determinants of expectation fulfilment

Younger age, higher preoperative mental wellbeing and preoperative function were predictive of better expectation fulfilment. Since patients with worse functional status had higher pre-THA expectations than surgeons [23], we had hypothesized that such patients had unrealistic expectations, which would not be entirely fulfilled. Our current findings confirm this hypothesis. Our previous study [23] also found that older individuals had less optimistic expectations. Their expectations might have still been over-rated, since the follow-up showed that expectations were less frequently fulfilled for older individuals. Preoperative patient education, which has been shown to modify expectations [25], should then target patients with higher risk of unmet expectations – ie older persons, with lower preoperative function or mental wellbeing [22].

However, fulfilment of expectations seemed to be predominantly associated with a better functional outcome, in accordance with previous literature findings [9,11,12,22]. Pain relief and limp absence were independent determinants of fulfilled expectations also. This is not surprising, as pain relief and improved mobility appear to be the most important preoperative expectations [22]. A residual limp could presumably influence postoperative function, but its independent association with the fulfilment score implies an additional mechanism. Mancuso et al. reported similar findings [13], and suggested that a residual limp may have an adverse psychological impact which could affect patients’ rating of their expectations’ fulfilment.

Both patients and surgeons had too optimistic expectations for certain activities, such as cutting toenails, putting on shoes, improving sexual activity, sport and professional activity. Previous studies [13,22] reported similar findings regarding the fulfilment of patients’ expectations. Surgeons need to be aware of such limits of THA, in order to inform their patients more precisely before surgery. Other expectations, such as relieving night pain, removing the need of a stick or medications, were frequently fulfilled for surgeons, but not for patients. This discrepancy could reflect an insufficiency in the preoperative patient-surgeon communication [38]. In the context of increasing litigation in medicine, the emphasis of preoperative counselling tends to address description of the possible risks of surgery, rather than on the expected outcomes and postoperative course [39].

### Strengths and limitations

In contrast to similar studies [9,13,22], the multi-centric design of this study aimed to optimize the external validity of results, although it was restricted to tertiary care centres. As satisfaction and expectation fulfilment differed according to study centres, mixed effect models were computed to adjust for centre effect, which was not addressed in previous multi-centric studies [18]. Another strength of this study is that missing data were scarce in comparison to other studies [6,11].

The main limitation of this work is the small number of events (10 patients dissatisfied), which could induce a risk of overfitting of predictive models [40]. However, we used a variable selection procedure, and our results were consistent with results from studies which were less prone to overfitting [9]. Another weakness of this study is that there is currently no validated approach to assess the fulfilment of expectations. Several authors used a non standardised retrospective assessment, with an important risk of recall bias [11,16]. Mancuso et al. [13], using the same questionnaire as we did, defined the fulfilment of expectations as the percentage of patients whose expectations were fulfilled completely in each domain. Our approach resembles the method recently described by Scott et al. [22], although it is unclear how unapplicable items were taken into account in their study.

## Conclusions

The main determinant of dissatisfaction 1 year after THA was the lack of fulfilment of patients’ expectations, independently of their preoperative level. Older age, worse mental wellbeing and disability were predictors of a poorer expectations’ fulfilment after surgery. After surgery, expectations fulfilment was mainly determined by postoperative function and pain relief. Surgeons had more reliable expectations and should better inform their patients of the expected outcomes, particularly regarding relieving night pain and removing the need of a stick. This study also highlights the urgent need to develop a valid tool to standardize the assessment of expectations and of their fulfilment.

## Competing interests

This study was supported by a grant from the Institut Fédératif de Recherche sur le Handicap (IFRH). No additional external funding was received. The funders had no role in study design, data collection and analysis, decision to publish, or preparation of the manuscript.

## Authors’ contributions

CJ, PR and SP conceived the study and its design. CP and CJ contacted patients, analyzed and interpreted the data. CP drafted the article. All authors provided critical input to drafts of the article and approved the final version.

## Pre-publication history

The pre-publication history for this paper can be accessed here:

http://www.biomedcentral.com/1471-2474/15/53/prepub
